# HLA-B*58:01 and Risk of Allopurinol-Induced Severe Cutaneous Adverse Reactions in the US

**DOI:** 10.1001/jamadermatol.2025.4240

**Published:** 2025-10-29

**Authors:** Chelsea N. Campbell, Matthew S. Krantz, Alexis Yu, Elizabeth J. Phillips

**Affiliations:** 1 Department of Pathology, Microbiology, and Immunology, Vanderbilt University Medical Center, Nashville, Tennessee; 2 Center for Drug Safety and Immunology, Department of Medicine, Vanderbilt University Medical Center, Nashville, Tennessee

## Abstract

**Importance:**

Carriage of HLA-B*58:01 has been shown to be associated with allopurinol-induced Stevens-Johnson syndrome and toxic epidermal necrolysis (SJS/TEN) and drug reaction with eosinophilia and systemic symptoms (DRESS) in many populations globally; however, there is a critical need to investigate whether this is generalizable to populations of mixed ancestry, such as those in the US.

**Objective:**

To assess the association of human leukocyte antigen (HLA) class I and II in a cohort of US patients who received a diagnosis of allopurinol-induced SJS/TEN or DRESS compared with allopurinol-tolerant and population control participants.

**Design, Setting, and Participants:**

This genetic association study included consenting individuals who had specialist-adjudicated allopurinol-induced SJS/TEN or DRESS (collectively allopurinol-induced severe cutaneous adverse reactions [SCARs]) between January 1, 2015, and December 31, 2024. HLA carriage in these individuals was compared with allopurinol-tolerant and population control participants identified through the Vanderbilt University Medical Center biobank, which includes 94 489 individuals with imputed HLA class I and II typing from genotyping array data. Data were analyzed from January 2025 to August 2025.

**Main Outcomes and Measures:**

The main outcome measure was the association of HLA class I and II alleles with allopurinol-induced SCARs. HLA class I and II conditional logistic regression case-control analyses were performed between patients with allopurinol-induced SCARs and both population control and allopurinol-tolerant control participants matched on age, sex, and self-identified race. Odds ratios (ORs) and 95% CIs were reported, with Bonferroni-corrected *P* < .05.

**Results:**

This genetic association study used conditional logistic regression analyses and included 16 patients with allopurinol-induced SCAR (mean [SD] age, 61.1 [12.6] years; 9 female patients [56.25%] and 7 male patients [43.75%]) and 160 allopurinol-tolerant control participants matched 10:1. Two HLA class I alleles were found to be independently associated with increased risk of allopurinol-induced SCAR: HLA-B*58:01 (OR, 28.0 [95% CI, 8.6-100.6]) and HLA-A*34:02 (OR, 20.6 [95% CI, 3.3-131.1]). No HLA class II alleles meeting the Bonferroni-corrected *P* < .05 level of significance were identified.

**Conclusions and Relevance:**

These findings suggest that although HLA-B*58:01 was found to be associated with allopurinol-induced SCARs, generalizing findings from previous studies, the allele was absent in more than one-third of the patient cohort and is therefore an incomplete indicator of risk. Importantly, in the US allopurinol-induced SCAR cohort, HLA-A*34:02 was found to be a second independent genetic risk factor for allopurinol-induced SCARs. These findings underscore the need to conduct population-based studies that both reproduce known and uncover novel HLA associations to reduce harm through contributions to screening, risk stratification, and diagnosis.

## Key Points

**Question:**

Is the association between HLA-B*58:01 and allopurinol-induced severe cutaneous adverse reactions (SCARs) generalizable to admixed populations in the US, or are additional HLA associations involved?

**Findings:**

In this genetic association study of 16 patients with adjudicated allopurinol-induced Stevens-Johnson syndrome and toxic epidermal necrolysis or drug reaction with eosinophilia and systemic symptoms from a heterogenous US-based population, an association with the established risk allele HLA-B*58:01 was demonstrated, and HLA-A*34:02 was identified as an additional independent risk factor.

**Meaning:**

These finding suggest that, although HLA-B*58:01 was found to be associated with allopurinol-induced SCARs, the allele was absent in more than one-third of the US patient cohort and is therefore an incomplete indicator of risk.

## Teaser

This genetic association study examined human leukocyte antigen (HLA) class I and II in a cohort of US patients who received a diagnosis of allopurinol-induced Stevens-Johnson syndrome and toxic epidermal necrolysis or drug reaction with eosinophilia and systemic symptoms compared with allopurinol-tolerant and population control participants.

## Introduction

Severe cutaneous adverse reactions (SCARs) are T-cell–mediated reactions to medications resulting in significant morbidity and mortality globally. Prevalent SCAR phenotypes include Stevens-Johnson syndrome and toxic epidermal necrolysis (SJS/TEN) and drug reaction with eosinophilia and systemic symptoms (DRESS). One of the most common drugs implicated in both SJS/TEN and DRESS is the uric acid–lowering agent, allopurinol. Although carriage of HLA-B*58:01 has been reported to contribute to 100% of patients with allopurinol-induced SCARs in Han Chinese populations,^1^ this is not true in Northern and Southern European populations, where only 55% to 64% of participants carried HLA-B*58:01.^2,3^ Despite having both an increased risk of developing SJS/TEN^4^ and a higher carriage rate of HLA-B*58:01 among US Black individuals, human leukocyte antigen (HLA) associations with allopurinol-induced SCARs in heterogenous populations in the US have not been defined.

## Methods

### Vanderbilt University Medical Center Biobank Cohorts

Allopurinol-tolerant control participants were selected from the Vanderbilt University Medical Center biobank (BioVU)^5^ population based on availability of the Expanded Multi-Ethnic Array (Infinium) typing, self-reported race, being 18 years of age or older at the time of allopurinol treatment, having baseline creatine values available in the 30 days prior to allopurinol initiation, having received 90 days or more of allopurinol treatment, the absence of any *International Classification of Diseases* codes pertaining to an allopurinol adverse reaction, and the absence of an allopurinol-specific allergy label. A 10:1 patient to control match by age, sex, and self-reported race was performed through optimal pair matching using the MatchIt R package.^6^ This study was approved by the institutional review board of Vanderbilt University Medical Center. All participants provided written informed consent. This study is reported following the Strengthening the Reporting of Genetic Association Studies (STREGA) reporting guideline.

### Patients With Allopurinol-Induced SCARs

Patients (n = 14) with specialist-adjudicated allopurinol DRESS and SJS/TEN were prospectively recruited between 2015 and 2024. Specialist-adjudicated patients with SJS/TEN (n = 2) who had developed SJS/TEN between 2009 and 2017 and participated from 2020 to 2024 were identified as part of a cross-sectional study of SJS survivors.

### HLA Typing

HLA class I and II alleles from BioVU participants were imputed from the Expanded Multi-Ethnic Array using SNP2HLA to 4-digit resolution.^7^ High-resolution HLA typing (HLA-A, HLA-B, HLA-C, HLA-DP, HLA-DQ, and HLA-DR) of patients with allopurinol-induced SCARs was performed using Illumina MiSeq as previously described.^8^

### Statistical Analysis

The stepwise conditional logistic regression of HLA class I and II alleles comparing patients with allopurinol-induced SCARs with allopurinol-tolerant control participants who were matched 10:1 was performed with the MiDAS HLA package, version 1.11.1 (R, version 4.4.2).^9^ HLA class I and II alleles meeting the unadjusted *P* < .05 along with their odds ratios (ORs), 95% CIs, and Bonferroni-corrected *P* values were reported. For alleles with Bonferroni-corrected *P* < .05, we further compared patients with allopurinol-induced SCARs with BioVU control participants, including self-identified Black, self-identified White, and overall populations by using the Fisher exact test, and reported ORs, 95% CIs, and *P* values. To evaluate for the gene-dose association of HLA-B*58:01, we performed the Fisher exact test and calculated ORs and 95% CIs with the Haldane^10^ modification for heterozygous and homozygous genotypes. Data were analyzed from January to August 2025.

## Results

A total of 16 patients (9 female [56.3%] and 7 male patients [43.8%]; mean [SD] age, 61.1 [12.6] years) were identified with allopurinol-induced SCARs who underwent high-resolution HLA typing (eTable 1 in Supplement 1) where a total of 11 patients (69.0%) self-identified as Black and 5 patients (31.0%) self-identified as White (Table 1). We identified 1122 allopurinol-tolerant control participants from BioVU (eFigure in Supplement 1) and subsequently identified 160 individuals matched (10:1) by age, sex, and race (eTable 2 in Supplement 1). In our case-control conditional logistic regression analysis of the allopurinol-induced SCAR and allopurinol-tolerant populations, we found 2 HLA class I alleles independently associated with increased risk of allopurinol-induced SCARs: HLA-B*58:01 (OR, 28.0 [95% CI, 8.6-100.6]; Bonferroni-corrected *P* < .001) and HLA-A*34:02 (OR, 20.6 [95% CI, 3.3-131.1]; Bonferroni-corrected *P* = .04) (Table 2). Although HLA-B*35:01 (OR, 10.7 [95% CI, 1.7-88.7]; Bonferroni-corrected *P* = .68) and HLA-B*58:02 (OR, 32.0 [95% CI, 1.1-1054.0]; Bonferroni-corrected *P* > .99) had an unadjusted *P* < .05 (Table 2), these alleles did not meet the Bonferroni-corrected *P* value level of significance. We did not identify any HLA class II alleles meeting the unadjusted *P* value or Bonferroni-corrected *P* value levels of significance. The percentage of individuals who carried HLA-B*58:01 (Figure, A) was higher among the patients with allopurinol-induced SCARs (62.5%; 10 of 16 patients) compared with the allopurinol-tolerant matched control participants (5.6%; 9 of 160 patients) (*P* < .001) and the BioVU overall population (2.3%; 2162 of 94 489 participants) (*P* < .001). The percentage of individuals who carried HLA-A*34:02 (Figure, B) was higher among the patients with allopurinol-induced SCARs (18.8%; 3 of 16 patients) compared with the allopurinol-tolerant matched control participants (4.4%; 7 of 160 participants) (*P* = .05) and the BioVU overall population (1.3%; 1246 of 94 489) (*P* = .001). Homozygosity at HLA-B*58:01 was associated with a higher risk of developing allopurinol-induced SCARs (OR, 55.3 [95% CI, 2.5-1208.6]) compared with heterozygosity in HLA-B*58:01 (eTable 3 in Supplement 1).

**Table 1.** Summary of the Allopurinol-Induced SCAR Case Cohort Demographic Characteristics, HLA Risk Allele Carriage, and Clinical Characteristics

Patient ID	Phenotype^a^	Sex	Race	HLA-B*58:01	Age, y	Latency period^b^	Dose at reaction, mg	BSA, %		SCORTEN	ALDEN score	RegiSCAR score	eGFR, mL/min/1.73 m^2^	Creatinine, mg/dL	History		SCAR clinical outcome
								Initial	Final						CKD	Gout	
1	SJS/TEN	F	Black	Positive	70s	23 d	U	30	70	4	6	NA	U	1.40	No	Yes	Survived
2	SJS/TEN	F	Black	Positive	80s	30 d	U	5	40	3	5	NA	23	2.04	Yes	No	Deceased
3	SJS/TEN	M	Black	Positive	50s	24 d	300	<10	>20	3	5	NA	15	4.13	Yes	Yes	Survived
4	SJS/TEN	F	Black	Negative	70s	2 d^c^	NA	15	18	4	4	NA	22	2.30	Yes	Yes	Survived
5	SJS/TEN	F	Black	Negative	50s	21 d	100, 3 times daily	10	76	5	6	NA	13	3.86	Yes	Yes	Survived
6	SJS/TEN	F	White	Positive	60s	14 d	U	U	U	U	U	NA	>60	0.61	No	Yes	Survived
7	SJS/TEN	M	White	Positive	70s	21 d	U	25	20	2	U	NA	>60	1.20	No	Yes	Survived
8	SJS/TEN	M	Black	Negative	40s	30 d	U	15	40	4	U	NA	>60	1.29	Yes	Yes	Survived
9	DRESS	F	Black	Negative	60s	14 d	100	NA	NA	NA	NA	5	8	5.37	Yes	Yes	Deceased
10	DRESS	F	Black	Positive	50s	42 d	100, twice daily	NA	NA	NA	NA	6	U	8.08	Yes	Yes	Survived
11	DRESS	M	Black	Positive	70s	30 d	100	NA	NA	NA	NA	4	46	1.51	No	Yes	Survived
12	DRESS	M	White	Negative	40s	U	NA	NA	NA	NA	NA	U	U	U	No	Yes	Survived
13	DRESS	F	White	Positive	60s	5 d	300	NA	NA	NA	NA	4	>60	0.73	No	Yes	Survived
14	DRESS	M	White	Positive	70s	21 d	300	NA	NA	NA	NA	3	U	U	No	Yes	Survived
15	DRESS	F	Black	Positive	40s	14 d	100, 3 times daily	NA	NA	NA	NA	4	23	2.57	Yes	Yes	Survived
16	DRESS	M	Black	Negative	50s	6 mo	300	NA	NA	NA	NA	6	6	9.79	Yes	Yes	Survived

Abbreviations: ALDEN, Algorithm of Drug Causality for Epidermal Necrolysis; BSA, body surface area; CKD, chronic kidney disease; DRESS, drug reaction with eosinophilia and systemic symptoms; eGFR, estimated glomerular filtration rate; F, female; HLA, human leukocyte antigen; ID, identifier; M, male; NA, not applicable; RegiSCAR, Registry of Severe Cutaneous Adverse Reactions; SCAR, severe cutaneous adverse reaction; SCORTEN, Severity-of-Illness Score for Toxic Epidermal Necrolysis; SJS/TEN, Stevens-Johnson syndrome and toxic epidermal necrolysis; U, unknown.

SI conversion factor: To convert creatinine to micromoles per liter, multiply by 88.4.

^a^ A positive diagnosis of SJS/TEN and DRESS and assignment of allopurinol as the culprit drug was obtained through specialist adjudication of the admission record. For patients with DRESS, a probability was assigned using the RegiSCAR scoring system. Scores in this system range from −4 to 9, where each risk factor adds 1 point to the score and higher scores indicate a higher probability of DRESS (scores <2 indicate no DRESS, 2-3 indicate possible DRESS, 4-5 indicate probable DRESS, and >5 indicate definite DRESS).

^b^ The number of days between the initiation of allopurinol treatment and the development of symptoms.

^c^ Developed SJS/TEN overlap on allopurinol rechallenge.

**Table 2.** Association of HLA-B*58:01 and HLA-A*34:02 With Allopurinol-Induced SCARs

Allele	Covariate^a^	Odds ratio (95% CI)	*P* value	Bonferroni-corrected *P* value
HLA-B*58:01	NA	28.0 (8.6-100.6)	<.001	<.001
HLA-A*34:02	HLA-B*58:01	20.6 (3.3-131.1)	<.001	.04
HLA-B*35:01	HLA-B*58:01 + HLA-A*34:02	10.7 (1.7-88.7)	.01	.68
HLA-B*58:02	HLA-B*58:01 + HLA-A*34:02 + HLA-B*35:01	32.0 (1.1-1054.0)	.03	>.99

Abbreviations: HLA, human leukocyte antigen; NA, not applicable; SCAR, severe cutaneous adverse reaction.

^a^ Conditional logistic regression analysis for HLA class I to compare patients with allopurinol-induced SCAR and allopurinol-tolerant (10:1) matched control participants (matched for age, sex, and self-identified race) performed stepwise with the addition of HLA class I allele covariates by highest significance (smallest *P* value) to each subsequent model to investigate HLA class I alleles that independently met *P* < .05 along with their Bonferroni-corrected *P* values.

**Figure.**
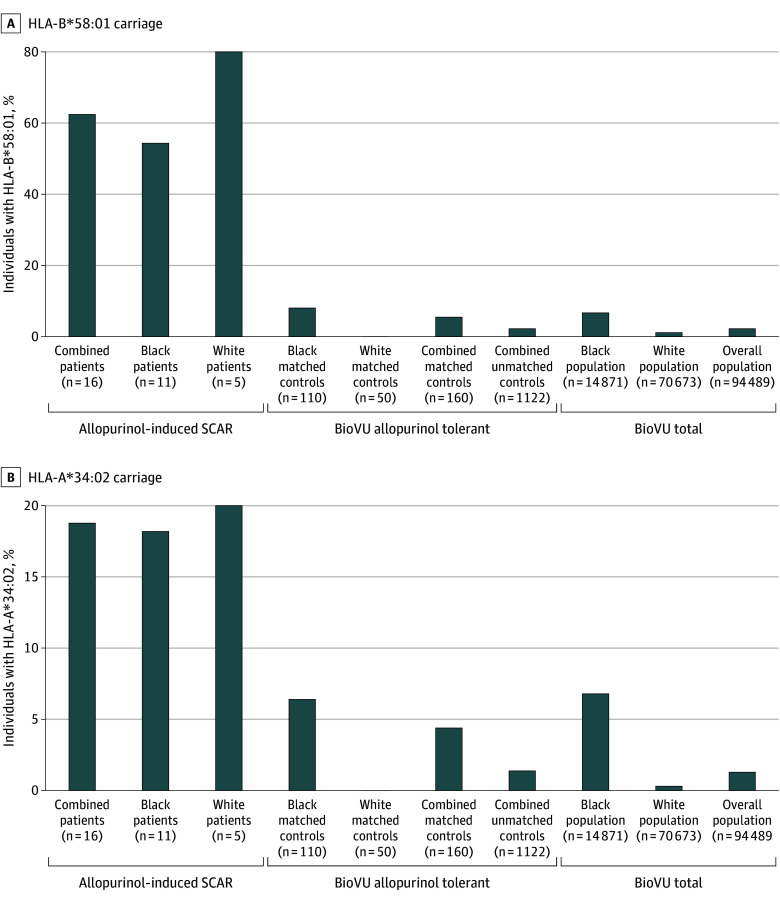
HLA-B*58:01 and HLA-A*34:02 Carriage Associated With Allopurinol-Induced Severe Cutaneous Adverse Reactions (SCARs) The *P* values for allele carriage rates among the combined patients with allopurinol-induced SCARs vs Vanderbilt University Medical Center Biobank (BioVU) allopurinol-tolerant control participants and the overall population in the BioVU total can be found in the Results.

## Discussion

Although HLA-B*58:01 has been identified as a risk allele for allopurinol-induced SCARs in multiple populations, to our knowledge, we are the first to demonstrate that HLA-B*58:01 is associated with allopurinol-induced SCARs in a heterogenous US-based population. Homozygosity at HLA-B*58:01 appeared to confer a particularly high risk, suggesting a potential gene-dose association. It is important to note, however, that HLA-B*58:01 was absent in more than one-third of our allopurinol-induced SCAR cohort overall as well as in 45% of self-identified Black patients, a population that is frequently prescribed allopurinol. Importantly, HLA-A*34:02 emerged as an additional risk factor for allopurinol-induced SCARs. Carriage of HLA-A*34:02 has previously been associated with allopurinol-induced, drug-induced liver injury, a condition with phenotypic overlap with DRESS,^11^ in which elevated liver function may occasionally be the first manifestation of DRESS, followed by skin involvement. Additionally, the HLA-A34 allele group, which includes HLA-A*34:02, was carried by 5 of 9 HLA-B*58:01–negative patients in a previously published allopurinol-induced SCAR cohort,^3^ further highlighting the potential significance of this allele in allopurinol-induced SCARs. We may have been underpowered to show a corrected association with HLA-B*35:01 (Table 2); however, future studies will be important to confirm whether this could be a true finding. From our prospective cohort, we also observed a single patient with allopurinol-induced SJS/TEN who carried HLA-B*58:02, an allele closely related to HLA-B*58:01 but differing by 3 amino acids in the peptide binding groove, which would not be detected by HLA-B*58:01–specific testing. Importantly, carriage of HLA-B*58:01, HLA-B*58:02, HLA-A*34:02, and HLA-B*35:01 accounted for 100% of patients with allopurinol-induced SCARs in the patient cohort. This finding suggests that multiple allele testing would more accurately identify representative risk in our US heterogenous population.^12^

### Limitations and Strengths

This study has some limitations. The sample size of this US allopurinol-induced SCAR cohort was small; however, its heterogeneity and distinct composition compared with previously studied populations underscore the significance and relevance of these findings and the strength of this study. The ability to investigate genetic associations, despite the small sample size and rarity of SCARs, reflects well on the high effect sizes. It is a notable and significant safety finding that our study suggests that screening for HLA-B*58:01 alone would miss more than one-third of patients with allopurinol-induced SCARs in US populations, including 45% of the US Black population where HLA-B*58:01 is carried at higher frequencies.

## Conclusions

In this genetic association study of US patients with allopurinol-induced SCARs, we replicated the established association of HLA-B*58:01 and identified HLA-A*34:02 as a novel risk factor for allopurinol-induced SCARs. A single-allele assay to investigate carriage of HLA-B*58:01 has become increasingly accessible, and prescreening prior to prescription is currently a selectively proposed mechanism to improve allopurinol safety.^13^ It is important to note, however, that an ideal screening test has 100% negative predictive value, and our US study identifies that carriage of HLA-B*58:01 alone is not capable of identifying all individuals at risk of allopurinol-induced SCARs. The absence of HLA-B*58:01 in more than one-third of the cohort and the identification of HLA-A*34:02 as an additional risk allele exemplify that population-based studies and multiallelic testing are needed to mitigate harm among those most vulnerable to these severe, life-threatening reactions.

## Notes

Supplement 1. **eFigure.** Consort diagram illustrating inclusion criteria for the BioVU overall population and allopurinol tolerant controls **eTable 1.** Complete HLA carriage of the allopurinol-SCAR cases cohort **eTable 2.** Characteristic summary of the allopurinol-SCAR cases and allopurinol tolerant controls from BioVU **eTable 3.** Homozygosity at HLA-B*58:01 increases risk of allopurinol-SCAR

Supplement 2. **Nonauthor collaborators.** Stevens-Johnson Syndrome/Toxic Epidermal Necrolysis (SJS/TEN) Survivor Study Collaborators

Supplement 3.
Data Sharing Statement
