# Supplementary material for: HLA-B*58:01 and Risk of Allopurinol-Induced Severe Cutaneous Adverse Reactions in the US
Source: JAMA Dermatol. 2025 Oct 29;161(12):1258–63. doi: 10.1001/jamadermatol.2025.4240 (PMC12573116; doi:10.1001/jamadermatol.2025.4240)
Supplement: Supplement 1. — eFigure. Consort diagram illustrating inclusion criteria for the BioVU overall population and allopurinol tolerant controls eTable 1. Complete HLA carriage of the allopurinol-SCAR cases cohort eTable 2. Characteristic summary of the allopurinol-SCAR cases and allopurinol tolerant controls from BioVU eTable 3. Homozygosity at HLA-B*58:01 increases risk of allopurinol-SCAR [file jamadermatol-e254240-s001.pdf]

Supplemental Online Content

Campbell CN, Krantz MS, Yu A, Phillips EJ; Stevens-Johnson Syndrome/Toxic Epidermal Necrolysis (SJS/TEN) Survivor Study Collaborators. HLA-B*58:01 and risk of allopurinol-induced severe cutaneous adverse reactions in the US. *JAMA Dermatol*. Published online October 29, 2025 doi:10.1001/jamaneurol.2024.3582

eFigure. Consort diagram illustrating inclusion criteria for the BioVU overall population and allopurinol tolerant controls.

eTable 1. Complete HLA carriage of the allopurinol-SCAR cases cohort

eTable 2. Characteristic summary of the allopurinol-SCAR cases and allopurinol tolerant controls from BioVU

eTable 3. Homozygosity at HLA-B*58:01 increases risk of allopurinol-SCAR

This supplemental material has been provided by the authors to give readers additional information about their work.

© 2025Campbell CN et al. *JAMA Dermatology*.

eFigure. Consort diagram illustrating inclusion criteria for the BioVU overall population and allopurinol tolerant controls.

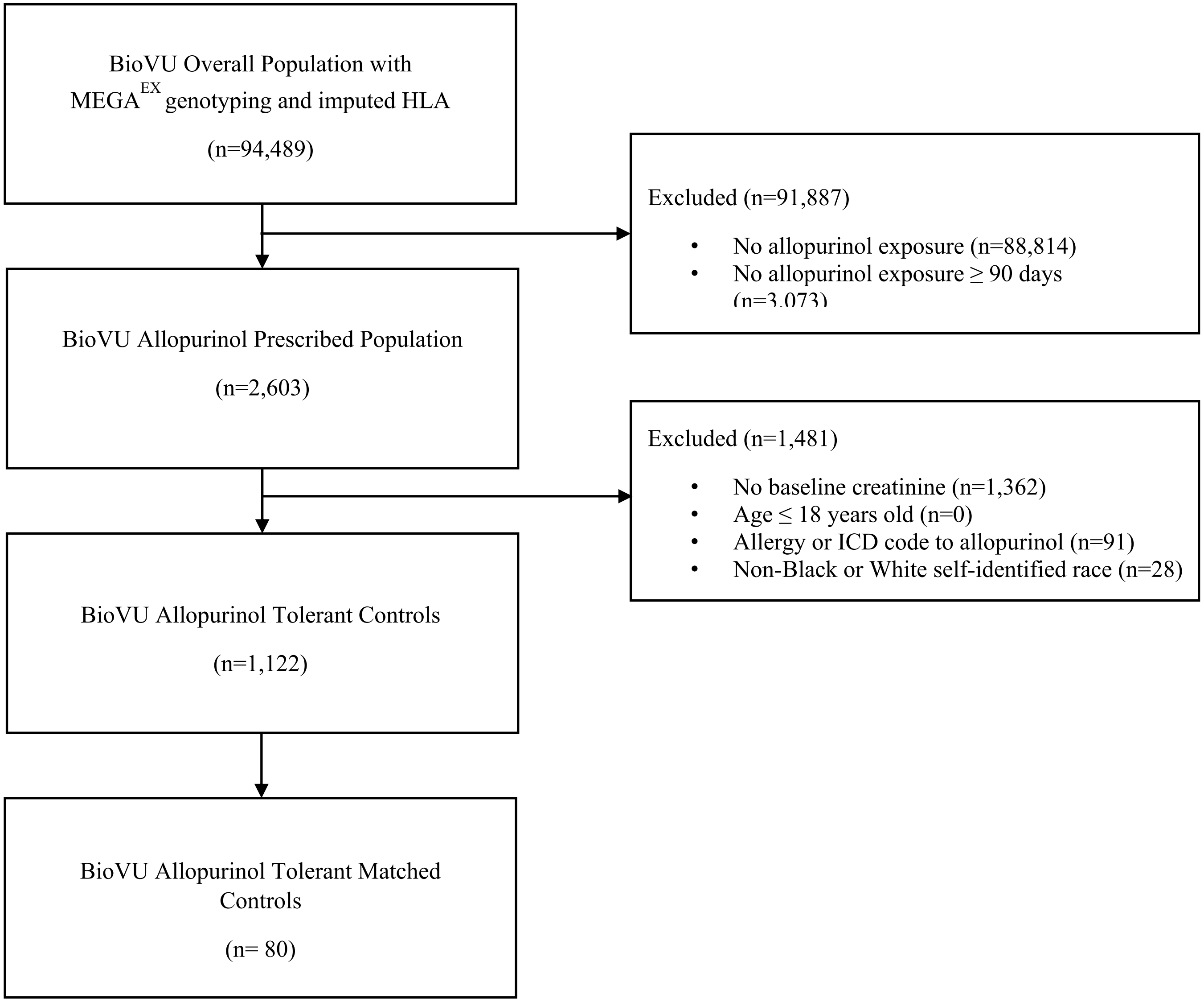

© 2025Campbell CN et al. *JAMA Dermatology*.

eTable 1. Complete HLA carriage of the allopurinol-SCAR cases cohort. High-resolution HLA typing was performed by Illumina MiSeq as previously published by sequencing of the Exon 2,3 region of HLA Class I and HLA-DQB1 and the Exon 2 region of HLA-DRB1, DQA1, and DPB1. Reference sequences were obtained through the IMGT/HLA Sequence Database and alleles were called through IIID HLA Analysis Suite. *NP,* allele not present.

	HLA-A		HLA-B		HLA-C		HLA-DPB1		HLA-DQA1		HLA-DQB1		HLA-DRB1		HLA-DRB3		HLA-DRB4		HLA-DRB5	
ID	Allele 1	Allele 2	Allele 1	Allele 2	Allele 1	Allele 2	Allele 1	Allele 2	Allele 1	Allele 2	Allele 1	Allele 2	Allele 1	Allele 2	Allele 1	Allele 2	Allele 1	Allele 2	Allele 1	Allele 2
1	29:02	68:02	15:03	58:01	2:10	07:01	01:01	11:01	01:02	02:01	02:02	06:02	07:01	15:03	NP	NP	01:01	NP	01:01	NP
2	02:02	02:02	58:01	58:01	08:02	08:02	01:01	04:02	01:01	03:01	03:03	05:01	13:01	14:01	02:02	02:02	NP	NP	NP	NP
3	23:01	30:01	42:01	58:01	07:01	17:01	01:01	01:01	04:01	05:01	02:01	04:02	03:01	03:01	01:01	02:02	NP	NP	NP	NP
4	29:02	74:01	35:01	53:01	04:01	07:01	02:01	17:01	01:02	04:01	03:01	05:01	08:04	13:02	03:01	NP	NP	NP	NP	NP
5	03:01	23:01	49:01	58:02	04:01	07:01	01:01	01:01	03:01	03:01	02:02	03:02	04:05	07:01	NP	NP	01:01	01:01	NP	NP
6	01:01	26:01	08:01	58:01	07:01	07:01	03:01	04:01	03:01	04:01	03:02	04:02	04:04	08:04	NP	NP	01:01	NP	NP	NP
7	03:01	25:01	07:02	58:01	03:02	07:02	04:01	14:01	01:02	01:02	06:02	06:09	13:02	15:01	03:01	NP	NP	NP	01:01	NP
8	30:01	30:02	35:01	42:01	04:01	17:01	02:01	04:02	02:01	04:01	02:02	04:02	03:02	07:01	01:01	NP	01:01	NP	NP	NP
9	23:01	34:02	35:01	44:02	04:01	05:01	04:01	04:02	01:02	03:01	03:01	06:02	04:01	11:01	02:02	NP	01:01	NP	NP	NP
10	23:01	74:01	58:01	58:01	03:02	07:01	01:01	18:01	01:02	02:01	02:02	06:02	07:01	11:01	02:02	NP	01:01	NP	NP	NP
11	30:02	36:01	13:02	58:01	08:04	16:01	02:01	13:01	01:02	04:01	04:02	06:02	08:04	15:03	NP	NP	NP	NP	01:01	NP
12	01:01	34:02	14:02	35:01	04:01	08:02	04:02	04:02	01:01	01:01	05:01	05:01	01:01	01:02	NP	NP	NP	NP	NP	NP
13	11:01	25:01	18:01	58:01	05:01	07:01	04:01	04:01	01:02	03:01	03:02	06:02	04:01	15:01	NP	NP	01:01	NP	01:01	NP
14	02:05	03:01	13:02	58:01	06:02	07:01	04:01	17:01	02:01	02:01	02:02	02:02	07:01	07:01	NP	NP	01:01	01:01	NP	NP
15	23:01	33:03	15:16	58:01	07:01	14:02	01:01	17:01	02:01	02:01	02:02	02:02	07:01	07:01	NP	NP	01:01	01:01	NP	NP
16	23:01	34:02	14:03	44:03	07:01	08:02	02:01	03:01	01:01	01:02	05:01	06:09	10:01	13:02	03:01	NP	NP	NP	NP	NP

© 2025Campbell CN et al. *JAMA Dermatology*.

eTable 2. Characteristic summary of the allopurinol-SCAR cases and allopurinol tolerant controls from BioVU. [1]Median (Q1, Q3); n (%)

		Allopurinol Tolerant Controls from BioVU	
**Variable**	**Allopurinol SCAR** N = 16[1]	**Matched** N = 160[1]	**Unmatched** N = 1,122[1]
**Age**	64 (52, 71)	63 (52, 71)	65 (57, 73)
**Gender**			
Female	9 (56%)	87 (54%)	342 (30%)
Male	7 (44%)	73 (46%)	780 (70%)
**Self-reported Race**			
Black	11 (69%)	110 (69%)	212 (19%)
White	5 (31%)	50 (31%)	910 (81%)
**HLA-B*58:01 Positive**	10 (62.5%)	9 (5.6%)	26 (2.3%)
**HLA-A*34:02 Positive**	3 (18.8%)	7 (4.4%)	16 (1.4%)

[1] Median (Q1, Q3); n (%)

© 2025Campbell CN et al. *JAMA Dermatology*.

eTable 3. Homozygosity at HLA-B*58:01 increases risk of allopurinol-SCAR. Fisher's exact test and calculated OR and 95% CI with Haldane's modification for heterozygosity and homozygosity at HLA-B*58:01 to evaluate gene-dose effect.

Genotype	OR (95% CI)	*P*
B*58:01 Heterozygous	28 (8.6, 100.6)	<.001
B*58:01 Homozygous	55.3 (2.5, 1208.6)	.01

© 2025Campbell CN et al. *JAMA Dermatology*.
