# Supplementary material for: HLA-B*58:01 and Risk of Allopurinol-Induced Severe Cutaneous Adverse Reactions in the US
Source: JAMA Dermatol. 2025 Oct 29;161(12):1258–63. doi: 10.1001/jamadermatol.2025.4240 (PMC12573116; doi:10.1001/jamadermatol.2025.4240)
Supplement: Supplement 2. — Nonauthor collaborators. Stevens-Johnson Syndrome/Toxic Epidermal Necrolysis (SJS/TEN) Survivor Study Collaborators [file jamadermatol-e254240-s002.pdf]

Supplemental Online Content: Nonauthor Collaborators
*First name, last name, and suffix (if applicable) are required and will appear in PubMed.

*Group Name(s): Stevens-Johnson Syndrome/Toxic Epidermal Necrolysis (SJS/TEN) Survivor Study Collaborators							
***First Name and Middle Initial(s)**	***Last Name**	***Suffix (eg, Jr, III)**	Academic Degrees	Institution	Location (city, state/province, country)	Role or Contribution, eg, chair, principal investigator	Group (if more than 1 Group listed in the byline) and/or Subgroup (eg, Steering Committee)
Roni	Dodiuk Gad		MD	Technion Institute of Technology	Haifa, Israel	Investigator/Adjudicator	SJS Survivor Study
Aaron	Drucker		MD	Women's college Hospital	Toronto, ON, Canada	Investigator/Adjudicator	SJS Survivor Study
Elizabeth	Ergen		MD	University of Tennessee	Knoxville, TN, USA	Investigator/Adjudicator	SJS Survivor Study
Rama	Gangula		MS	Vanderbilt University Medical Center	Nashville, TN, USA	Assistant	SJS Survivor Study
Michelle	Goh		MBBS	Austin Health	Heidelberg, VIC, Australia	Investigator/Adjudicator	SJS Survivor Study
Benjamin	Kaffenberger		MD	The Ohio State University Wexner Medical Center	Columbus, OH, USA	Investigator/Adjudicator	SJS Survivor Study
Dana	King		BS	Vanderbilt University Medical Center	Nashville, TN, USA	Assistant	SJS Survivor Study
Rebecca	Lee		MPH	Vanderbilt University Medical Center	Nashville, TN, USA	Assistant	SJS Survivor Study
Kelby	Mahan		LPN	Vanderbilt University Medical Center	Nashville, TN, USA	Assistant	SJS Survivor Study
Michelle	Martin-Pozo		PhD	Vanderbilt University Medical Center	Nashville, TN, USA	Assistant	SJS Survivor Study
Robert	Micheletti		MD	University of Pennsylvania	Philadelphia, PA, USA	Investigator/Adjudicator	SJS Survivor Study
April	O'Connor		RN	Vanderbilt University Medical Center	Nashville, TN, USA	Assistant	SJS Survivor Study
Amy	Palubinksy		PhD	Vanderbilt University Medical Center	Nashville, TN, USA	Assistant	SJS Survivor Study
Suman	Pakala		ME	Vanderbilt University Medical Center	Nashville, TN, USA	Assistant	SJS Survivor Study

Supplemental Online Content: Nonauthor Collaborators
*First name, last name, and suffix (if applicable) are required and will appear in PubMed.

***First Name and Middle Initial(s)**	***Last Name**	***Suffix (eg, Jr, III)**	Academic Degrees	Institution	Location (city, state/province, country)	Role or Contribution, eg, chair, principal investigator	Group (if more than 1 Group listed in the byline) and/or Subgroup (eg, Steering Committee)
Elizabeth	Williams		MPH	Vanderbilt University Medical Center	Nashville, TN, USA	Assistant	SJS Survivor Study
Kristina	Williams		RN	Vanderbilt University Medical Center	Nashville, TN, USA	Assistant	SJS Survivor Study
