# Supplementary material for: HLA-B*58:01 and Risk of Allopurinol-Induced Severe Cutaneous Adverse Reactions in the US
Source: JAMA Dermatol. 2025 Oct 29;161(12):1258–63. doi: 10.1001/jamadermatol.2025.4240 (PMC12573116; doi:10.1001/jamadermatol.2025.4240)
Supplement: Supplement 3. — Data Sharing Statement [file jamadermatol-e254240-s003.pdf]

Data Sharing Statement

Campbell. HLA-B*58:01 and Risk of Allopurinol-Induced Severe Cutaneous Adverse Reactions in the US. *JAMA Dermatol*. Published October 29, 2025. doi:10.1001/jamadermatol.2025.4240

Data
Data available: Yes
Data types: Deidentified participant data
How to access data: Please direct requests to the corresponding author, Dr. Elizabeth J. Phillips, at elizabeth.j.phillps@vumc.org
When available: With publication

Supporting Documents
Document types: None

Additional Information
Who can access the data: De-identified clinical data will be made available to all investigators.
Types of analyses: Data for high-resolution HLA sequencing will be made available to anyone requesting the data for any purpose or analysis.
Mechanisms of data availability: High resolution HLA sequencing is available in the e-repository of this manuscript.
